# Effects of social economics changes on children health status in Indonesia (IFLS 1993 – 2007)

**DOI:** 10.1186/1471-2458-14-S1-P3

**Published:** 2014-01-29

**Authors:** Vissia Didin Ardiyani

**Affiliations:** 1 Health Polytechnic of Palangka Raya, Indonesia

## Background

Stunting in Indonesia is still high. In 1992 there were 50% of children aged less than 5 years classified as stunted. This situation remained until 1997. Stunting is the result of complex interactions involving environmental factors and rapid socio-economic changes have been associated with the improvement in stunting.

## Materials and methods

In this study we examined the improvement in stunting and associated socio-economic changes that have occurred in Indonesia from 1993 – 2007. This study used longitudinal data from Indonesian Family Life Surveys in 1993 through 2007. Data were analysed using a Generalised Estimating Equation (GEE).

## Results

Results showed that there were socio-economic changes in household on average 11% per year in the middle quintile. There were association between socio-economic increase and the decrease of stunting prevalence (P = 0.000; OR = 1.6; 95% CI = 1.251-2.017). Most of the decrease in rates of stunting among children occurred in the wealthiest fifth. In 1997 the likelihood of stunting increased 5% per year in the bottom fourth and fifth of the population. After 1997, there was a decreasing trend. This study also found that stunting in children who were exposed to the care of midwife during childhood (1993) was significantly better than that of their peers in the same age and cohort in communities without midwives (P = 0.001; OR = 1.6; 95% CI = 1.177-1.817).

## Conclusions

The increase of socio-economic status in the household can improve stunting. In addition, this study explained that midwives were playing important role in children’s height. However, there are some limitations in the present study. The variable of empowering programme was not included in the analysis and thus it was not known whether socio-economic changes were due to empowering programme or not.

